# Impact of Pneumonia on Cognitive Aging: A Longitudinal Propensity-Matched Cohort Study

**DOI:** 10.1093/gerona/glac253

**Published:** 2022-12-16

**Authors:** Merle K Hendel, Debora Rizzuto, Giulia Grande, Amaia Calderón-Larrañaga, Erika J Laukka, Laura Fratiglioni, Davide L Vetrano

**Affiliations:** Aging Research Center, Department of Neurobiology, Care Sciences and Society, Karolinska Institutet and Stockholm University, Stockholm, Sweden; Aging Research Center, Department of Neurobiology, Care Sciences and Society, Karolinska Institutet and Stockholm University, Stockholm, Sweden; Gerontology Research Center, Stockholm, Sweden; Aging Research Center, Department of Neurobiology, Care Sciences and Society, Karolinska Institutet and Stockholm University, Stockholm, Sweden; Aging Research Center, Department of Neurobiology, Care Sciences and Society, Karolinska Institutet and Stockholm University, Stockholm, Sweden; Gerontology Research Center, Stockholm, Sweden; Aging Research Center, Department of Neurobiology, Care Sciences and Society, Karolinska Institutet and Stockholm University, Stockholm, Sweden; Gerontology Research Center, Stockholm, Sweden; Aging Research Center, Department of Neurobiology, Care Sciences and Society, Karolinska Institutet and Stockholm University, Stockholm, Sweden; Gerontology Research Center, Stockholm, Sweden; Aging Research Center, Department of Neurobiology, Care Sciences and Society, Karolinska Institutet and Stockholm University, Stockholm, Sweden; Gerontology Research Center, Stockholm, Sweden

**Keywords:** Cognitive decline, Cohort study, Dementia, Pneumonia, Propensity score

## Abstract

**Background:**

Acute clinical events, such as pneumonia, may impact physical functionality but their effect on cognition and the possible duration of this effect remains to be quantified. This study investigated the impact of pneumonia on cognitive trajectories and dementia development in older people.

**Methods:**

Data were obtained from 60+ years old individuals, who were assessed from 2001 to 2018 in the population-based SNAC-K study (Sweden). Participants were eligible if they were not institutionalized, had no dementia, and did not experience pneumonia 5 years prior to baseline (*N* = 2 063). A propensity score was derived to match 1:3 participants hospitalized with a diagnosis of pneumonia (*N* = 178), to nonexposed participants (*N* = 534). Mixed linear models were used to model cognitive decline. The hazard of dementia, clinically diagnosed by physicians following Diagnostic and Statistical Manual of Mental Disorders (DSM)-IV, was estimated using Cox regression models.

**Results:**

We found a transient impact of pneumonia on cognitive decline in the first 2.5 years (*B* = −0.94, 95% confidence interval [CI] −1.75, −0.15). The hazard ratio (HR) for dementia was not statistically significantly increased in pneumonia participants (HR = 1.17, 95%CI 0.82, 1.66).

**Conclusions:**

The transient impact of pneumonia on cognitive function suggests an increased need of health care for patients after a pneumonia-related hospitalization and reinforces the relevance of pneumonia prevention.

Cognitive aging describes the process of gradual, longitudinal changes in cognitive function observed during the aging process (1). It is determined by several predisposing (2) and contextual factors acting throughout the life course (3) and shows wide interindividual differences in the rate of cognitive decline (4). The cognitive decline accelerates with age (5,6). After 70, the risk of developing dementia increases significantly (7). So far, there is no pharmacological treatment available that can cure dementia or counteract the progression of cognitive impairment. Therefore, investigating modifiable risk factors is key to better understand the etiology of dementia and to pave the way toward novel preventive strategies.

Acute clinical events, such as major infections, have a dramatic impact on individuals’ health (8) and might constitute turning points in the process of cognitive aging (9). Pneumonia frequently occurs in older people and requires hospitalization among severe cases (10), and the recent COVID-19 pandemic has further highlighted its dramatic impact on survival and health (11). In addition to its relation to physical impairments and mortality (12), pneumonia has been associated with impaired cognitive function. A systematic review from our group identified several studies that investigated the association of pneumonia and cognitive function (13). Although most of these studies agree on the possible association between pneumonia and cognitive decline and dementia, only 2 addressed dementia, and there is only a single study investigating cognitive decline and dementia together. Potential weaknesses of these prior studies are that they do not implement methods of causal inference that try to minimize risks of reverse causality and residual confounding, often at play when studying pneumonia and cognition. Furthermore, they are not always performed on population-based cohorts, which may introduce a selection bias. Characterizing the changes in cognition associated with pneumonia is crucial to examine pneumonia as a potentially modifiable risk factor for cognitive decline and possible treatment options.

The overall aim of this study was to investigate the impact of pneumonia on cognitive trajectories in older adults. Specifically, we aimed to (i) examine the association of pneumonia with cognitive decline and dementia and (ii) investigate how the association developed over time (short-term vs long-lasting effect).

## Method

### Study Design, Participants, and Procedure

**Figure 1.**
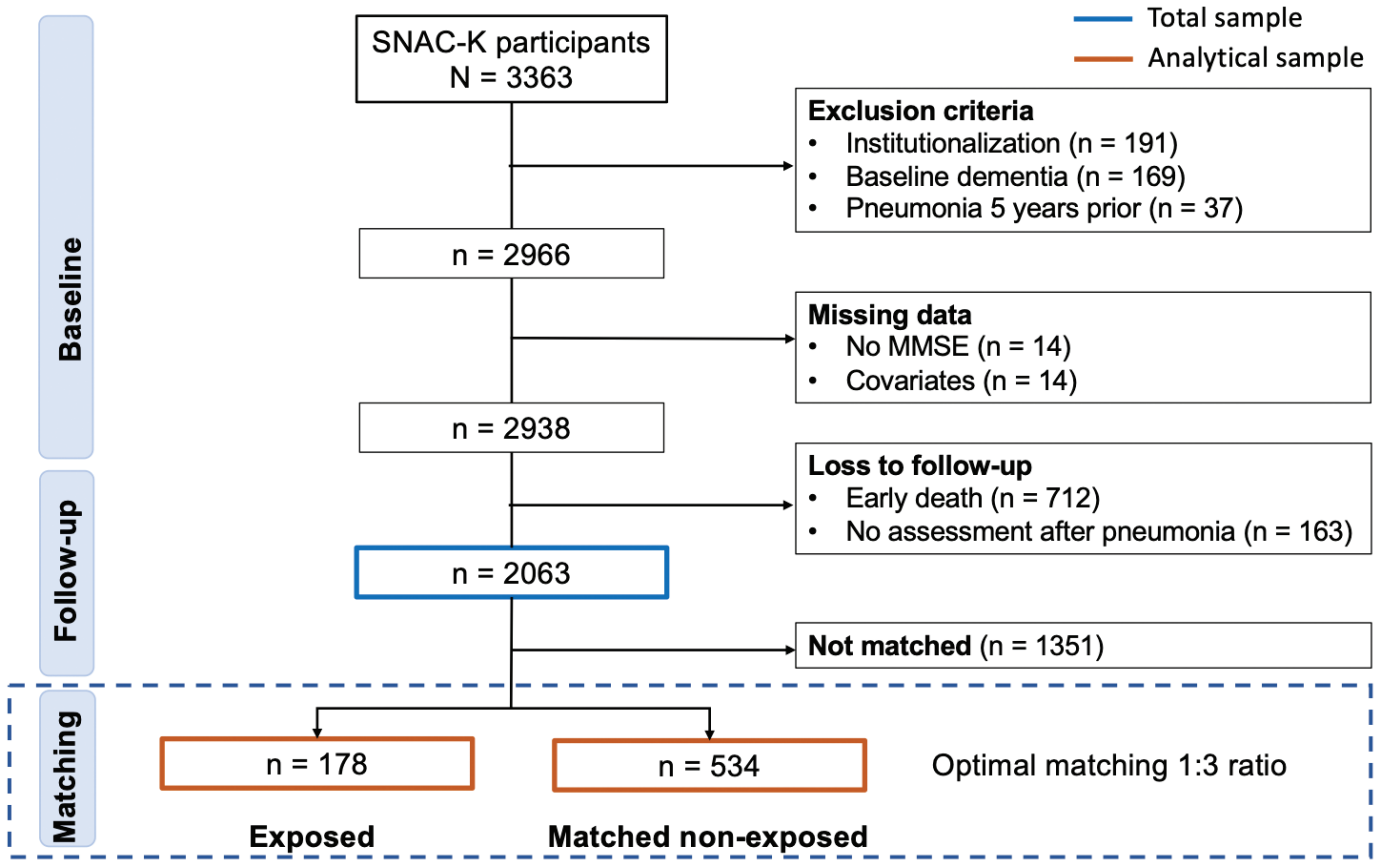
Flow chart of the identification of the 2 sample populations: a total sample and an analytical sample.

This study derived data from the SNAC-K cohort, Swedish National Study on Aging and Care in Kungsholmen (14) (https://www.snac-k.se/). SNAC-K is an ongoing longitudinal population-based cohort study including 3 363 participants ≥60 years at baseline (2001–2003) living in the Kungsholmen district of Stockholm, Sweden. Assessments take place every 6 years for participants <72 years old and every 3 years for those ≥78. Fifteen years of follow-up data were used in this study; participants were examined extensively by nurses, physicians, and psychologists between 2001 and 2018. All participants gave written informed consent; the Regional Ethical Review Board in Stockholm, Sweden, approved the SNAC-K protocols. Following pre-established criteria, we excluded 1 300 participants (Figure 1) due to institutionalization (*n* = 191); a diagnosis of dementia at the baseline assessment (2001–2003; *n* = 169); and a history of pneumonia 5 years prior to the baseline assessment (*n* = 37). Further, 712 participants died after the baseline assessment, 163 participants with pneumonia did not have a cognitive assessment after the acute event, and 28 missed a cognitive assessment at baseline or relevant variables to calculate the propensity score. The total sample resulted in 2 063 individuals from whom we derived a propensity score (see below), of which 712 were matched and included in the analysis. Participants with dementia (*n* = 38) at the index date were excluded in a sensitivity analysis.

### Measurements

#### Pneumonia detection

The following International Classification of Diseases, tenth revision (ICD-10) codes were used to identify hospitalizations due to pneumonia: J09-J18 and J20-J21. The events were derived from the National Patient Register covering all hospital admissions in Sweden since 1969, which has been shown to be a valid measure to derive pneumonia diagnoses (15). Participants experiencing a first-ever unplanned pneumonia-related hospitalization from 2001 onwards were defined as exposed participants, and the date of the first occurrence of pneumonia was considered the index date.

#### Outcomes

Cognitive performance was measured at each assessment by means of the Mini-Mental State Examination (MMSE) (16). Dementia was defined as a global decrement in cognitive functioning to the extent of affecting one’s independence in the activities of daily living. The clinical diagnosis of dementia was carried out by SNAC-K physicians according to the DSM-IV criteria following a 3-step procedure. Two clinicians made independent preliminary diagnoses. In case of a discordant diagnosis, a senior neurologist was consulted for a third opinion. For participants who died before a follow-up assessment without having received a dementia diagnosis, dementia was ascertained using hospital record, hospital discharge registers, and death certificates. Cognitive status and dementia, as well as all other covariates, were assessed at baseline and throughout the course of the study at every assessment, keeping with the SNAC-K design.

#### Covariates

Individual factors (ie, age, sex, education) as well as contextual factors (ie, living situation, civil status) were evaluated. Educational attainment was categorized as elementary, high school, and university or higher. Civil status was categorized into married, widowed or divorced, and single. Alcohol intake was divided into 3 categories: no or occasional, light to moderate (1–7 drinks/wk for women, 1–14 drinks/wk for men), and heavy (≥8 drinks/wk for women, ≥15 drinks/wk for men). Smoking was categorized as never, former, and current smoking. Body mass index was calculated by dividing weight in kilograms by height in square meters. Level of disability was measured as the sum of the basic and instrumental activities of daily living participants were unable to perform independently. The total number of medications (coded in accordance with the ATC classification) at the time of assessment was also considered. Clinical diagnoses were made by SNAC-K physicians based on self-reports of the participants, medical charts, patient registers, anamnestic details, clinical examination, and proxy information to increase diagnostic reliability. Walking speed was assessed using a 6-m path and by asking participants to walk at their usual speed. The last obtained values for the respective variables prior to pneumonia hospitalization were considered for matching participants using propensity scores. DNA was extracted from peripheral blood samples, apolipoprotein E (*APOE*) alleles were genotyped. *APOE* allelic status was dichotomized in ε4 carriers and ε4 noncarriers. The number of hospitalizations unrelated to pneumonia was extracted from the National Patient Register. Dates of death between 2001 and 2019 were obtained from the Swedish Cause of Death Register.

### Analytical Approach

Using the information from the National Patient Register, the hospital admission date for the first pneumonia occurrence was detected and used as the index date. A dummy event date was attributed to each participant not experiencing pneumonia. The date was randomly generated in between the baseline and last assessment for each participant without pneumonia. Index dates for participants with pneumonia and participants without pneumonia were centered to allow for comparability (Supplementary Figure 1). The current study utilized matched propensity score analysis with linear mixed-effect models implemented in Stata 16.0 (StataCorp LLC, College Station, TX) for Microsoft Windows and R Statistics (R x64 3.6.3) for Mac OS in which the significance level was set to α = 0.05. Data were expressed as means (±standard deviation [*SD*]) for continuous variables and frequencies (%) for categorical variables.

#### Matching procedure

The propensity score represents a person’s probability of experiencing pneumonia based on the specified covariates and offers a nonparametric matching approach. It enables accounting for characteristics that are assumed to be different in individuals with and without pneumonia (17). This procedure allowed us to reduce the risk of confounding by matching the propensity scores of the two groups a priori as closely as possible in order to study the effect of pneumonia in isolation. This accounts for characteristics that may predispose participants both to the pneumonia occurrence as well as to cognitive decline. After the identification of pneumonia and no pneumonia participants, the propensity score for each individual was derived using the last information available before the index date. Specifically, this includes the last available information for all pneumonia participants at the time point throughout the course of the study before a participant was hospitalized for pneumonia or, in the case of a no-pneumonia patient, before the dummy date. The propensity score was generated via logistic regression models and based on a selection of demographic (ie, age, sex, education, marital status, and smoking), clinical (ie, the number of hospitalizations, MMSE, incident dementia, hypertension, heart failure, atrial fibrillation, ischemic heart disease, stroke, chronic kidney disease, and cancer), and functional (walking speed, limitations in basic, and instrumental activities of daily living) characteristics. Covariates were selected based on clinical expertise—that is, factors impacting cognitive function and showing an association with pneumonia—as well as the best data fit with the highest accuracy (area under the curve = 0.79) and parsimony. The matching was carried out in a 3:1 ratio (no pneumonia:pneumonia), utilizing an optimal matching procedure (18), in which people with a pneumonia were matched to 3 clinical counterparts with the closest propensity score value. Once chosen, participants without pneumonia could not be matched again. Different matching techniques were tested in sensitivity analyses to ensure robust results.

#### Exploring possible associations

Upon verifying that no assumptions were violated (ie, linearity between model residuals, homogeneity of residual variance across groups, normality of residuals), maximum likelihood multiple linear mixed regression analyses with random intercept and slope were performed to investigate the differences in cognitive trajectories between individuals with and without pneumonia, prior and after the event. Locally weighted scatterplot smoothing (Lowess) functions were used to identify turning points in cognitive trajectories for both groups. The association between pneumonia hospitalizations and incident dementia was tested by means of Cox regression models. The interaction terms between a number of covariates (age, sex, and median propensity score) and the exposure were entered and tested in the models. All models were adjusted by age, sex, education, and the number of hospitalizations unrelated to pneumonia.

#### Sensitivity analyses

We repeated the analysis after excluding participants who developed dementia during the observation period before the pneumonia event. Stratified analyses were carried out accordingly by age, sex, and median propensity score level.

## Results

**Figure 2.**
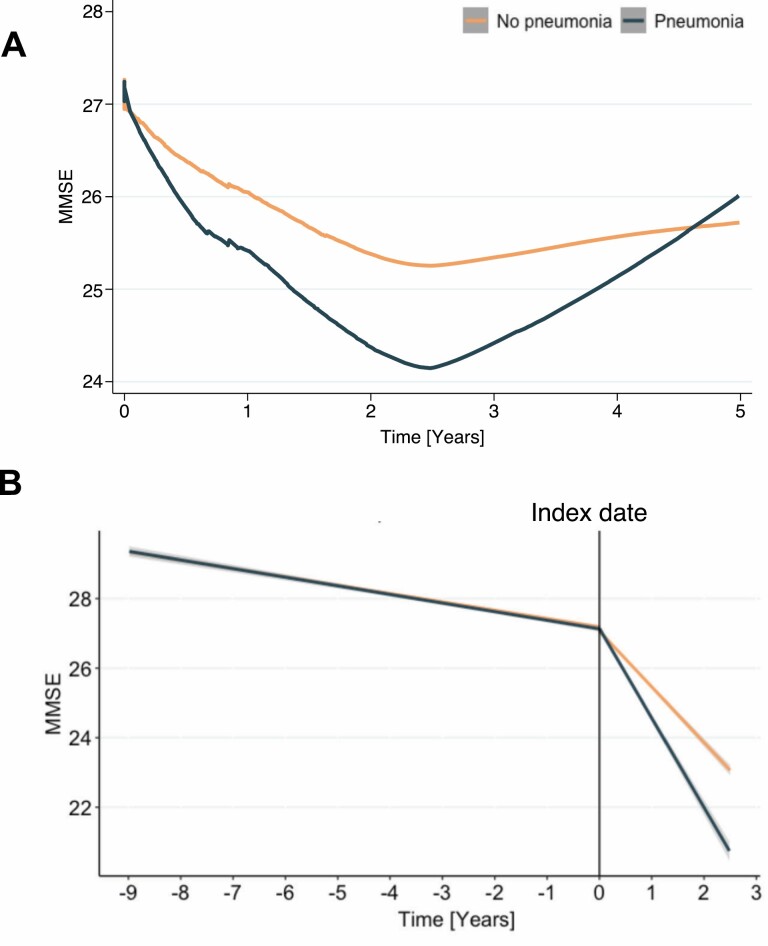
Cognitive trajectories in relation to pneumonia occurrence. (**A**) A Lowess smoothing function was applied in the first years after the index date (ie, time of pneumonia hospitalization) in order to identify turning points. The visual inspection implied a possible short-term effect of pneumonia on cognition. (**B**) Short-term effects were investigated in the first 2.5 years following pneumonia hospitalization. Participants experiencing pneumonia showed a significantly steeper decline in cognitive function compared to their clinical counterparts not experiencing pneumonia (*B* = −0.95, 95% CI −1.75, −0.15; *p* = .020). Models were adjusted for age, sex, education, and number of hospitalizations.

During the observation period (2001–2018), out of 2 063 individuals from the total sample, 178 participants were hospitalized due to pneumonia. Based on their propensity score, people hospitalized for pneumonia were matched with 534 participants with no pneumonia, resulting in an analytical sample of 712 participants (Figure 1). On average, our sample was 81.27 years old (*SD* = 9.33 years), and 62.36% were females and had an average of 1.14 disabilities (*SD* = 2.17). SNAC-K participants who experienced a pneumonia but were excluded from the analysis due to missing assessments after the hospitalization presented with a lower age (74.51 vs 78.63 years, *p* < .001), were less frequently female (52.15% vs 64.66%) and had a similar disability burden (0.70 vs 1.76, *p* = .791). Clinical and functional characteristics of the 2 samples (total and analytical) can be found in Table 1. In the total sample, several characteristics, including age, education, and several clinical and functional measures were distributed differently between participants with and without pneumonia (*p* < .05). After matching, except for the number of hospitalizations, none of these differences remained statistically significant in the analytical sample (*p* for all >.05). Other matching techniques (ie, nearest neighbor, matching with/without replacement) resulted in the same levels of characteristic balancing (data not shown). The analysis of changes in cognitive trajectories prior to and after the index date was restricted to an observation period of 16 years (9 years prior to 7 years after, ie, years with at least 50 MMSE observations) to ensure robust results (Supplementary Figure 2). During this period, there were a total of 2 963 MMSE individual measurements (ie, 755 in pneumonia participants and 2 208 in no pneumonia participants). According to results from adjusted linear mixed regression models, the rate of MMSE change was not different across the 2 groups during the 9 years before the index date (*B* = −0.01 per year, 95% confidence interval [CI] −0.07, 0.05; *p* = .772; Figure 2).

**Table 1.** Characteristics at the Closest Assessment Before Pneumonia Hospitalization in the Total Sample and in the Analytical Sample Before and After Matching

	Total Sample*			Analytical Sample^†^			
	Pneumonia *n* = 178	No pneumonia *n* = 1899	*p*	Pneumonia *n* = 178	No pneumonia *n* = 534	*p*	Whole sample *n* = 712
Age (mean, *SD*)	81.27 (9.33)	73.85 (10.21)	**<.001**	81.27 (9.33)	81.57 (8.84)	.697	81.50 (8.95)
Sex (females, %)	62.36	64.93	.492	62.36	64.23	.653	63.76
Education (%)			**<.001**			.919	
Elementary	21.91	12.20		21.91	23.22		22.89
High school	48.88	48.59		48.88	47.57		47.89
University	29.21	39.20		29.21	29.21		29.21
Marital status (%)			**.003**			.979	
Married	42.13	48.81		42.13	40.64		41.01
Widow/Divorced	45.51	34.91		45.51	48.12		47.47
Unmarried/Living alone	12.36	16.24		12.36	11.23		11.52
Smoking (%)			.807			.965	
Current/former	55.05	51.51		55.05	53.93		54.21
Never	44.38	47.90		44.38	45.13		44.94
Alcohol consumption			.188			.297	
Heavy	13.48	18.41		13.48	15.54		15.03
Never/occasionally	32.58	28.12		32.58	37.27		36.10
BMI (mean, *SD*)	25.62 (3.71)	25.87 (3.96)	.429	25.62 (3.71)	25.62 (3.83)	.978	25.62 (3.80)
Walking speed in m/s (mean, *SD*)	0.78 (0.41)	1.03 (0.43)	**<.001**	0.78 (0.41)	0.78 (0.43)	.936	0.78 (0.43)
ADL+IADL disabilities (one or more, %)	41.01	23.24	**<.001**	41.01	41.01	1.000	41.01
MMSE (mean, *SD*)	27.57 (3.18)	28.42 (2.48)	**<.001**	27.57 (3.18)	27.62 (3.00)	.870	27.61 (3.04)
Hypertension (%)	85.39	75.12	**.002**	85.39	84.46	.764	84.69
Heart failure (%)	20.79	7.43	**<.001**	20.79	17.04	.260	17.98
Ischemic heart disease (%)	26.40	12.15	**<.001**	26.40	24.35	.617	25.00
Atrial fibrillation (%)	23.03	7.90	**<.001**	23.03	19.85	.363	20.65
Depression (%)	10.67	10.56	.961	10.67	11.99	.637	11.66
Stroke (%)	18.54	6.63	**<.001**	18.54	16.48	.526	16.99
Chronic kidney disease (%)	53.37	29.60	**<.001**	53.37	51.31	.634	51.83
Solid neoplasms (%)	21.35	13.21	**.003**	21.35	18.54	.410	19.24
Hematological neoplasms (%)	2.25	0.6	**.028**	2.25	1.69	.628	1.83
COPD (%)	13.48	9.92	.134	13.48	11.80	.552	12.22
Dementia (%)	5.62	2.39	**.011**	5.62	5.43	.924	5.48
Number of drugs (mean, *SD*)	6.12 (3.97)	3.97 (3.32)	**<.001**	6.12 (3.97)	5.81 (3.61)	.341	5.89 (3.70)
Number of hospitalizations (mean, *SD*)	2.30 (2.82)	0.87 (1.57)	**<.001**	2.30 (2.82)	1.79 (2.24)	**<.014**	1.92 (2.41)
APOE E4 allele (%)	21.14	28.69	**.034**	21.14	24.61	.351	23.73

*Note*s: BMI = Body mass index; COPD = Chronic obstructive pulmonary disease; MMSE = Mini-Mental State Examination; ADL = Activities of daily living; IADL = Instrumental activities of daily living. Differences between groups were determined using t-tests for continuous variables and Chi-square tests for categorical variables. Significant differences are displayed in bold. Missing information: Marital status: *N* = 6 (0.84%); alcohol consumption: *N* = 348 (48.88%).

*Used to derive the propensity score.

^†^Obtained by matching cases of pneumonia with people without pneumonia using the propensity score.

The long-term association between pneumonia and cognition was studied throughout the 7 years following pneumonia hospitalization: the rate of MMSE change was not different across the 2 groups (*B* = −0.28 per year, 95%CI −0.65, 0.10; *p* = .146). When applying the Lowess function, a steep decline in MMSE scores was observed only in participants experiencing pneumonia during the first 2.5 years of follow-up, which was followed by an inversion of the curve after 4.5 years, resembling the MMSE levels of the individuals with no pneumonia hospitalization (Figure 2A). After restricting the multivariate analysis to the first 2.5 years, a steeper MMSE decline was observed in participants with pneumonia (*B* = −0.95 per year, 95%CI −1.75, −0.15; *p* = .020; Figure 2B).

When excluding 39 participants that had developed dementia by the time of pneumonia occurrence, the effect of pneumonia on MMSE change over 2.5 years of follow-up was slightly attenuated, yet statistically significant (*B* = −0.82 per year, 95%CI −1.63, −0.10; *p* = .046). No additive interactions were detected between MMSE change and sex, age, or median propensity score. Consistently, no differences were detected across sex, age, and propensity score strata.

After excluding 38 individuals with dementia at the index date, the incidence rate of dementia in 7 years was 46/1 000 person-years in participants with pneumonia and 40/1 000 person-years in their matched counterparts. According to adjusted Cox regression models, the hazard for dementia was not statistically significantly increased for participants with versus without pneumonia (hazard ratio [HR] = 1.17, 95%CI 0.82, 1.66; *p* = .358). To note, over the same period, the death hazard was not increased in participants who experienced pneumonia, compared to their counterparts (HR = 1.06, 95%CI 0.84, 1.34; *p* = .647).

## Discussion

In this propensity score-matched cohort study, against our expectation, we found that older adults hospitalized for pneumonia experience only a transient acceleration in cognitive decline. More specifically, pneumonia was independently associated with a drop in MMSE of more than 2 points during the first 2.5 years, with a subsequent recovery and no increased risk of dementia. To the best of our knowledge, this is the first study undertaking a longitudinal analysis of cognitive changes before and after pneumonia hospitalization utilizing a rigorously matched comparison group and investigating the temporal dynamics of this phenomenon.

Cognitive impairment and dementia are common findings in older adults and predispose for the development of pneumonia (19). Thus, ruling out reverse causality when investigating the impact of pneumonia on cognition represents a major challenge. Previous population-based studies investigating the association between pneumonia and cognitive aging overall agree on the link between pneumonia and cognitive impairment (9,20–23) or increased dementia incidence (24,25). In these studies, different measures of cognitive function and dementia have been used, ranging from remote proxies, such as telephone interviews, to validated neuropsychological tests, such as MMSE. Compared to ours, no study had a pre- and post-pneumonia follow-up period as long as 15 years, with some studies being limited to less than a year or unspecified time periods (20–22). Most studies did not utilize a propensity score matching for a more rigorous group comparability (9,21,22,24) and if so, mostly only accounted for demographic variables (20,23), which neglects the clinical heterogeneity of older people experiencing pneumonia. Although most studies were of a prospective nature, information about the period prior to the hospitalization was not incorporated (9,21–23,25). Altogether, these issues limit the possibility to rule out residual confounding and reverse causality in these studies.

The absence of a long-lasting impact of pneumonia on cognitive decline and incident dementia is a crucial finding of this study as it contradicts previous research (9,24,25). The study design and methodology implemented aimed to account for residual confounding, reduce risks of reverse causality, and quantify temporal dynamics between pneumonia and cognitive decline. The propensity score analysis not only incorporated sociodemographic information but also several clinical and functional variables. The aligned cognitive trajectories before hospitalization between exposed versus nonexposed participants indicated that they showed comparable cognitive status at that time point, as well as similar trajectories until that moment.

A transient brain damage could be explained by several underlying biological mechanisms triggered by pneumonia, including systemic inflammation, hypoxia, and hypoperfusion. Pneumonia is accompanied by the systemic inflammatory response (26,27). Systemic inflammation is associated with acute psychological changes, including depressive symptoms, delirium, and reduced cognitive performance (28–33). The pneumonia-associated alveolar damage may impair gas exchanges, leading to respiratory insufficiency and eventually systemic hypoxia (34). Brain hypoxia has been associated with subcortical brain damage and mild cognitive impairment, mainly characterized by impairments in attention, executive functions, and processing speed (35). Furthermore, hypoxic stimuli were shown to accelerate the deposition of beta-amyloid in the brain, even if this is less in line with the transient impact of pneumonia on cognitive function pointed out in our study (36). To note, a frequent occurrence in older adults experiencing pneumonia is a multi-organ decompensation, involving mainly the cardiovascular system. Previous studies observed an increased risk for cardiovascular events in pneumonia patients compared to controls, with a particularly high risk following the first year after the pneumonia hospitalization (37). Increased risks of heart failure (38), myocardial infarction (39), atrial fibrillation (40,41), and stroke (42) have been reported in older people after pneumonia episodes. These 3 conditions are well-known risk factors of cognitive decline (43,44).

Another possible mechanism underlying the observed association is that patients hospitalized for pneumonia are often bedridden (45). A frequently observed consequence of being bedridden is the “disuse syndrome.” It has been associated with reduced independence in activities of daily living and negative functional outcomes, which persist for a long time after discharge (46,47). The reduced physical activity levels usually observed as a consequence of hospitalizations further contribute to increase the risk of frailty and cognitive impairment (48). Another consequence of hypomobility might be a higher risk of thromboembolic events such as pulmonary embolism (49), thereby contributing to impair systemic oxygenation. Finally, the change in medication regimens occurring after a hospitalization episode, along with the increase in polypharmacy, might further partially explain the poorer cognitive performance observed in individuals experiencing pneumonia. It is well known that complex pharmacological regimens are associated with a higher anticholinergic burden and several side effects that may impact cognition (50,51).

Our study has implications not only for the understanding of the mechanisms contributing to cognitive decline in older people but also for identifying potential actions for its prevention. Since pneumonia mortality rates dramatically decreased throughout the 21st century, its impact on the persons suffering from it is mainly related to an accelerated health decline and complex long-term consequences (52). Findings from our study highlight the importance of effective interventions and care for patients experiencing pneumonia, particularly in the short-term after hospitalization for pneumonia, which can be summarized into the following points: (i) acute and post-acute care could improve the cognitive outcomes of patients, as more assistance might be needed during and following hospitalization (22); (ii) timely and rigorous rehabilitation might enable a prompt functional recovery after pneumonia (53,54); and (iii) to decrease the number of severe pneumonia cases, primary and secondary prevention strategies should be improved, including vaccinations. Pneumonia represents one of the leading causes for avoidable hospital admissions (55), and vaccinations against influenza and pneumococcus have shown to decrease pneumonia incidence, as well as its adverse impacts (56).

Some limitations of this study should be acknowledged. One major issue dominating longitudinal studies concerns the problem of reverse causality. Studying a phenomenon in isolation constitutes a major challenge as several other competing risk factors need to be taken into consideration. Thus, even though the propensity score in the current study included the most extensive selection of clinical, functional, and demographic characteristics so far, there might be other remaining unknown factors potentially involved in the association between pneumonia and cognitive function, as well as dementia (eg, genetic risk factors increasing the predisposition to both pneumonia and dementia or environmental risk factors such as air pollution). Our results can be extrapolated to pneumonia survivors only, as the participants who did not complete an assessment after hospitalization were not included in our analyses. Thus, our study might have suffered from survival bias and possibly been underpowered to detect long-term cognitive decline and incident dementia in pneumonia patients. However, we did not detect an excess of mortality in the pneumonia group, and the diagnosis of dementia also took the information into account reported in the death register. The attenuation of the association after excluding people with dementia suggests that the phenomenon might be accelerated in people with dementia. It has to be acknowledged that the MMSE as a cognitive measurement is a screening test aimed to assess global cognitive function. Thus, it is not suitable to provide information on performance in individual domains. The ceiling effects observed in this test might furthermore offer an explanation for the lack of long-term effects of pneumonia on cognitive function (57). An additional shortcoming of this study is the lack of knowledge about pneumonia etiology, severity, and received treatment. As a result, we were not able to investigate the impact of such features on the extent of cognitive decline and the risk of dementia. Lastly, the matching procedure resulted in comparable characteristics between pneumonia and no pneumonia participants prior to the pneumonia event. It is possible that the participants without pneumonia experienced a similar event, other than a pneumonia-related hospitalization, that impacts cognitive function and dementia incidence. Such acute clinical events could be: (i) less severe cases of pneumonia not requiring hospitalization or (ii) cardiovascular events that are also associated with pneumonia occurrence (37). Thus, it is possible that the effect of pneumonia on cognitive function was attenuated by our strict matching procedure. Nevertheless, we were able to detect an independent association between pneumonia and cognitive decline in the short-term.

In summary, by utilizing a propensity score analysis taking into account several demographic, clinical, and functional characteristics, as well as observations prior and after hospitalization, we were able to identify a transient impact of pneumonia on cognitive function in the first years after the infection. Conclusively, preventing pneumonia in the first place, and offering the best possible care for those hospitalized, might delay transitory cognitive decline.

## Supplementary Material

glac253_suppl_Supplementary_Material

## Data Availability

Data are from the SNAC-K project, a population-based study on aging and dementia (http://www.snac-k.se/). Access to these original data is available to the research community upon approval by the SNAC-K data management and maintenance committee. Applications for accessing these data can be submitted to Maria Wahlberg (Maria.Wahlberg@ki.se) at the Aging Research Center, Karolinska Institutet.
