# Supplementary material for: Impact of Pneumonia on Cognitive Aging: A Longitudinal Propensity-Matched Cohort Study
Source: J Gerontol A Biol Sci Med Sci. 2022 Dec 16;78(8):1453–60. doi: 10.1093/gerona/glac253 (PMC10395566; doi:10.1093/gerona/glac253)
Supplement: glac253_suppl_Supplementary_Material [file glac253_suppl_supplementary_material.pdf]

Supplementary material

Supplementary Table 1: *Results of the linear mixed model before the date of hospitalization for pneumonia*

				95% Confidence Interval		
	B	SE	z	LB	UB	p
Intercept	28.53	0.89	31.97	26.79	30.28	< 0.001
Pneumonia	-0.03	0.21	-0.16	-0.44	0.37	0.870
Time	-0.24	0.02	-14.48	-0.27	-0.21	< 0.001
Pneumonia * Time	-0.01	0.03	-0.29	-0.07	0.05	0.772
Age	-0.03	0.01	-2.76	-0.05	-0.01	0.006
Sex	-0.32	0.18	-1.78	-0.68	0.03	0.075
Education	0.58	0.12	4.70	0.34	0.82	< 0.001
Number of hospitalizations	-0.08	0.04	-2.23	-0.15	-0.01	0.026

Note: LB = Lower bound, UB = Upper bound

Supplementary Table 2: *Results of the linear mixed model investigating the long-term and short-term effects of cognition after hospitalization for pneumonia*

				95% Confidence Interval		
	B	SE	z	LB	UB	p
**Long-term effects (7 years follow-up)**						
Intercept	33.36	1.20	27.88	31.01	35.70	< 0.001
Pneumonia	-0.13	0.26	-0.48	-0.65	0.39	0.633
Time	-0.89	0.10	-9.28	-1.08	-0.70	< 0.001
Pneumonia * Time	-0.28	0.19	-1.45	-0.65	0.10	0.146
Age	-0.09	0.01	-6.74	-0.12	-0.06	< 0.001
Sex	-0.20	0.24	-0.81	-0.67	0.28	0.416
Education	0.62	0.15	3.86	0.31	0.94	< 0.001
Number of hospitalizations	-0.12	0.05	-2.52	-0.21	-0.03	0.012
**Short-term effect (2.5 years follow-up)**						
Intercept	30.17	1.13	25.52	29.96	34.39	< 0.001
Pneumonia	0.07	0.25	0.28	-0.42	0.56	0.780
Time	-1.55	0.20	-7.66	-1.94	-1.15	< 0.001
Pneumonia * Time	-0.95	0.41	-2.33	-1.75	-0.15	0.020
Age	-0.07	0.01	-5.78	-0.10	-0.05	< 0.001
Sex	-0.26	0.23	-1.15	-0.71	0.18	0.251
Education	0.57	0.15	3.73	0.27	0.87	< 0.001
Number of hospitalizations	-0.09	0.04	-2.15	-0.18	-0.01	0.031

Note: LB = Lower bound, UB = Upper bound

Supplementary Table 3: *Association between pneumonia hospitalization and dementia incidence*

	**IR**	**HR**
	per 1000/person year	(95% CI)
**Long-term (7 years)**		
No Pneumonia	40	1 (Ref.)
Pneumonia	46	1.17 (0.82, 1.66)
**Short-term (2.5 years)**		
No Pneumonia	71	1 (Ref.)
Pneumonia	74	0.97 (0.65, 1.48)

Note: IR = Incident rate, HR = Hazard Ratio, CI = Confidence interval

eFigure 2. Number of observations over time during the course of the study

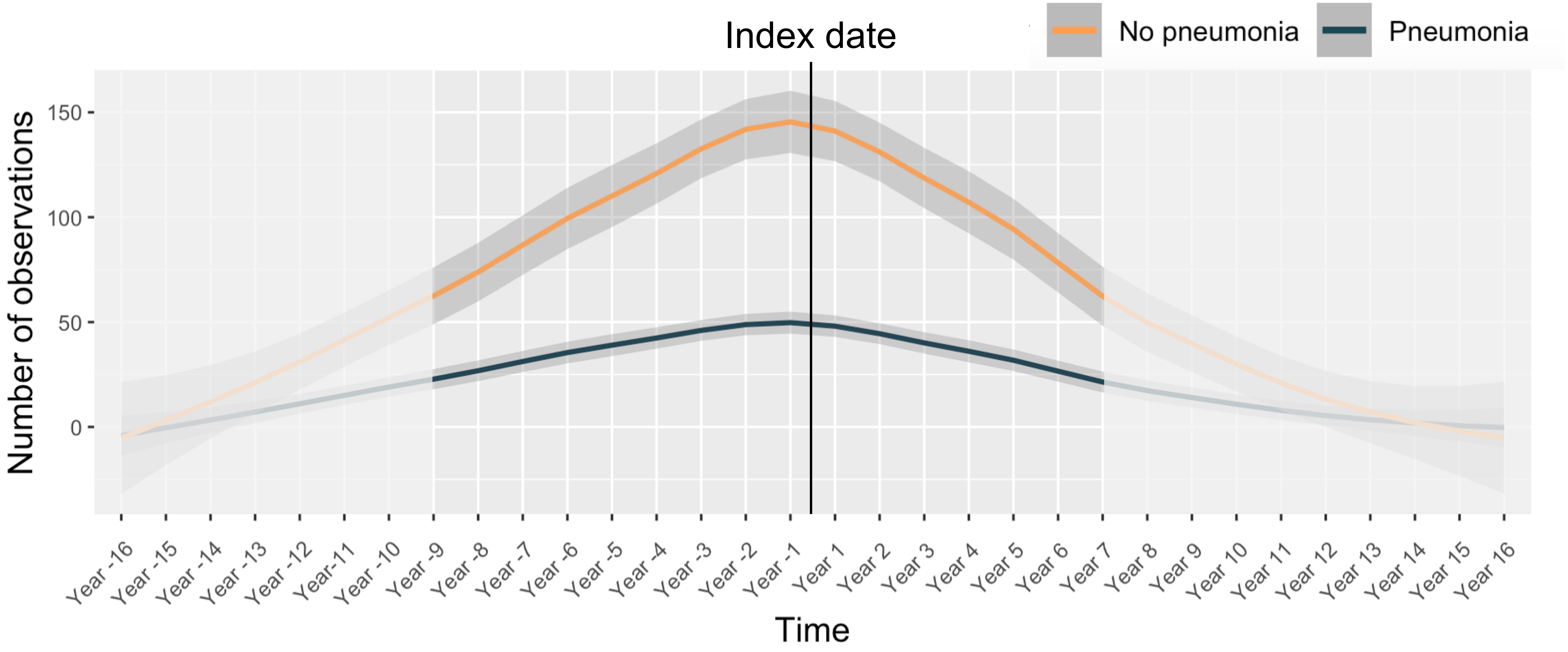

Note: Smoothed lines indicate the mean number of observations and. The analysis was restricted to nine years prior to the index date and seven years after the index date in order to have at least 50 observations per year.

eFigure 1. Centering of exposed and non-exposed participants

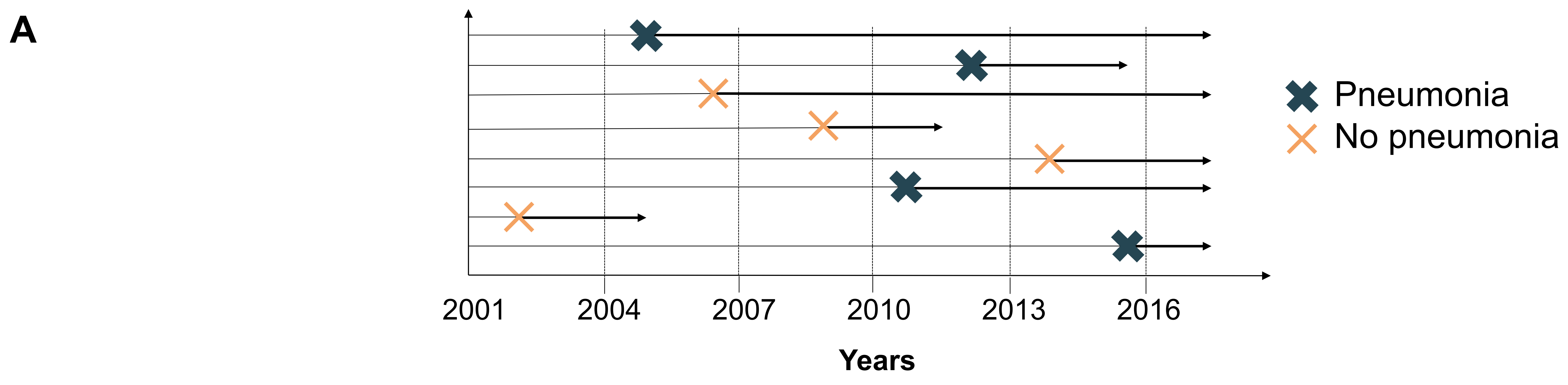

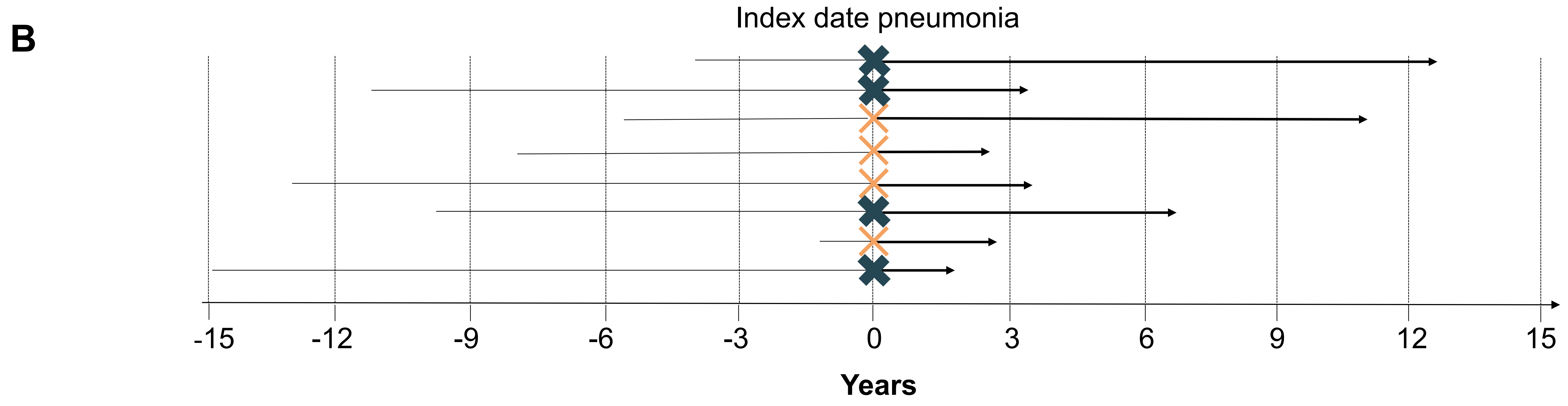

A. Pneumonia and no pneumonia participants at the beginning of the study. For pneumonia participants, the dates for pneumonia hospitalization were determined through the National Patient Register. For participants not experiencing pneumonia, dummy index dates were generated randomly in-between the baseline and last assessment for each participant. This ensured that both pneumonia and no pneumonia participants had at least one assessment before and one assessment after the index date. **B.** After the index date generation, the baseline time for every participant was centered around the index date. The index date was represented as time point 0. Time before the event was coded in negative numbers. Time from the index date onwards was coded in positive numbers.
